# Kisspeptin in the Prediction of Pregnancy Complications

**DOI:** 10.3389/fendo.2022.942664

**Published:** 2022-07-19

**Authors:** Jovanna Tsoutsouki, Bijal Patel, Alexander N. Comninos, Waljit S. Dhillo, Ali Abbara

**Affiliations:** Section of Endocrinology and Investigative Medicine, Imperial College London, Hammersmith Hospital, London, United Kingdom

**Keywords:** gestational trophoblastic disease (GTD), gestational diabetes mellitus (GDM), pre-ecalmpsia (PET), foetal growth restriction (FGR), hypertensive disorders of pregnancy (HDP), preterm (birth), miscarriage, kisspeptin

## Abstract

Kisspeptin and its receptor are central to reproductive health acting as key regulators of the reproductive endocrine axis in humans. Kisspeptin is most widely recognised as a regulator of gonadotrophin releasing hormone (GnRH) neuronal function. However, recent evidence has demonstrated that kisspeptin and its receptor also play a fundamental role during pregnancy in the regulation of placentation. Kisspeptin is abundantly expressed in syncytiotrophoblasts, and its receptor in both cyto- and syncytio-trophoblasts. Circulating levels of kisspeptin rise dramatically during healthy pregnancy, which have been proposed as having potential as a biomarker of placental function. Indeed, alterations in kisspeptin levels are associated with an increased risk of adverse maternal and foetal complications. This review summarises data evaluating kisspeptin’s role as a putative biomarker of pregnancy complications including miscarriage, ectopic pregnancy (EP), preterm birth (PTB), foetal growth restriction (FGR), hypertensive disorders of pregnancy (HDP), pre-eclampsia (PE), gestational diabetes mellitus (GDM), and gestational trophoblastic disease (GTD).

## Introduction

Kisspeptin is best known for its role as a hypothalamic neuropeptide that regulates gonadotrophin releasing hormone (GnRH) secretion (1). Indeed, early studies showed that inactivating variants of the kisspeptin receptor result in pubertal failure due to hypogonadotrophic hypogonadism, confirming the importance of kisspeptin signalling to reproductive health (2, 3).

During pregnancy, kisspeptin is produced in large amounts by the placenta and thus there is significant interest in evaluating its potential as a novel marker of pregnancy complications (4). Kisspeptin is a peptide encoded by the *KISS-1* gene that binds to a G-protein coupled kisspeptin receptor (*KISS-1R*, previously known as the orphan receptor GPR54) (5). Kisspeptin levels in the circulation are several hundred fold higher during healthy pregnancy compared to the non-pregnant state (6, 7). This review will summarise data evaluating kisspeptin’s role as a putative biomarker of pregnancy complications including miscarriage, ectopic pregnancy (EP), preterm birth (PTB), foetal growth restriction (FGR), hypertensive disorders of pregnancy (HDP), pre-eclampsia (PE), gestational diabetes mellitus (GDM), and gestational trophoblastic disease (GTD).

## Kisspeptin

The gene encoding kisspeptin (*KISS-1*) was first identified in 1996 as a metastasis tumour-suppressor gene in malignant melanoma cell lines and its peptide product was initially termed ‘metastin’ (8). Subsequently, it became known as kisspeptin in homage to its discovery in Hershey, Pennsylvania, USA, the hometown of the famous chocolate Hershey’s kisses (8). The *KISS-1* gene, located on chromosome 1q32, encodes a 145 amino acid prepropeptide that is post-translationally cleaved into biologically active kisspeptin peptides of different amino acid lengths indicated by their suffix: e.g. kisspeptin -54, -14, -13, and -10 (5, 9, 10). All of these peptides bind and activate the kisspeptin receptor through their shared C-terminal region decapeptide motif (Arg-Phe-NH_2_) (5, 10). Kisspeptin is expressed in multiple tissues including the hypothalamus, limbic system, gonads, pancreas, and liver, but is particularly abundant in the placenta, and thus is believed to play an important role in pregnancy (10, 11).

## Kisspeptin in Healthy Pregnancy

**Figure 1:**
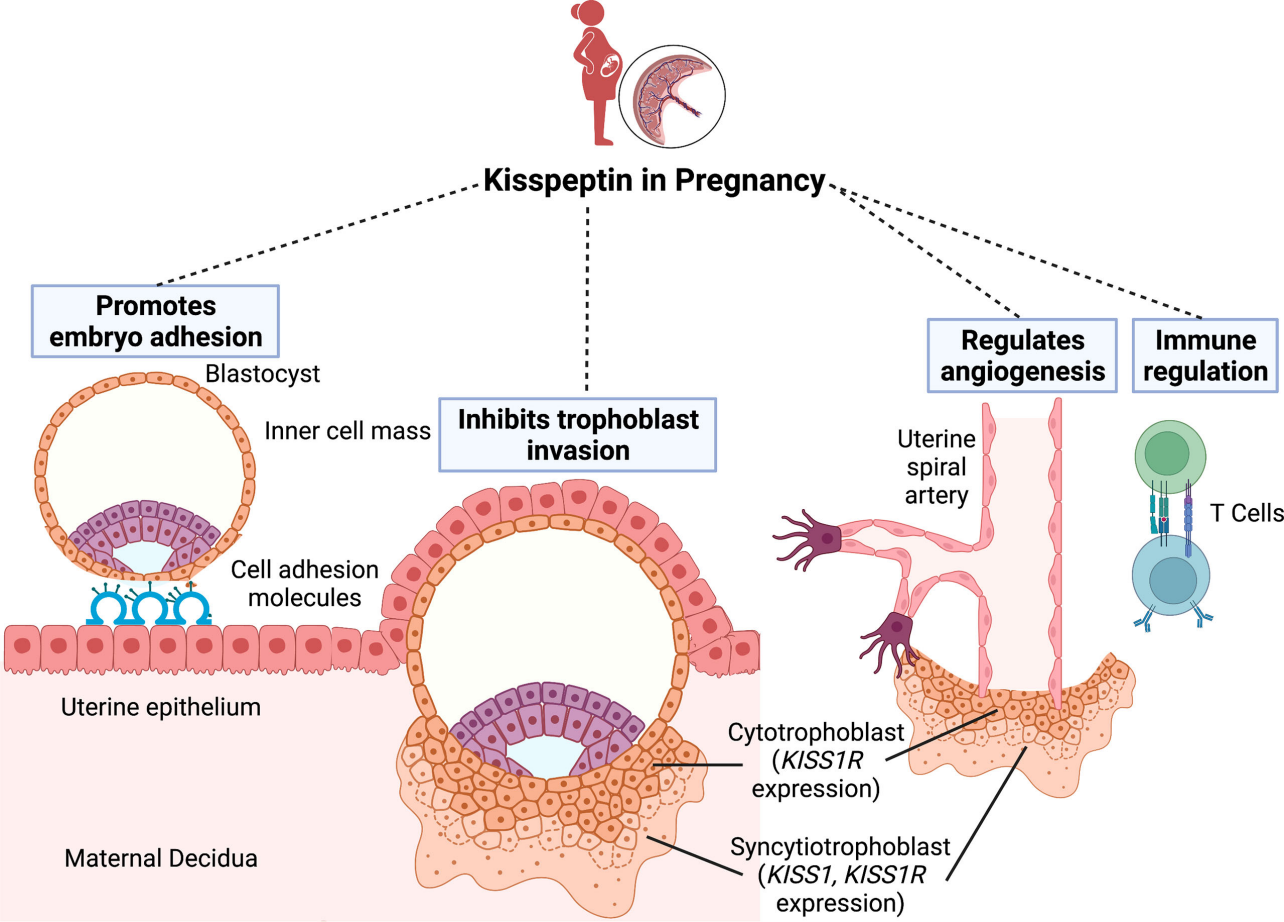
The role of kisspeptin in embryo implantation. Successful implantation requires communication between the blastocyst and a receptive uterine epithelium. Kisspeptin initially promotes embryo attachment to the endometrial epithelium through interaction with cell adhesion molecules. Once the blastocyst penetrates the epithelium, the trophoblast cells differentiate into the inner cytotrophoblast and outer syncytiotrophoblast cells. Whilst the cytotrophoblast cells express the kisspeptin receptor (*KISS-1R*), the syncytiotrophoblast cells express both *KISS-1R* and the kisspeptin gene (*KISS-1*). Kisspeptin subsequently regulates implantation by inhibiting excessive trophoblast invasion into the endometrium. Finally, kisspeptin also has roles in uterine spiral artery remodelling and immune regulation to avoid maternal foetal rejection. Figure created with BioRender.com.

Kisspeptin plays a key role in implantation and decidualisation. Kisspeptin promotes embryo attachment to the endometrium through interaction with cell adhesion molecules, and stimulates stromal decidualisation by up-regulating leukaemia inhibitory factor (LIF) (12) (**Figure 1**). Kisspeptin also attenuates the excessive migration and invasion of trophoblasts through inhibition of the matrix metalloproteinases (MMP) 2 and 9 (13–15). Kisspeptin may also impact angiogenesis and uterine spiral artery modelling (16–18). A further relevant mechanism of kisspeptin in pregnancy relates to the maternal immune tolerance needed to avoid foetal rejection. Indeed, *in vitro* incubation with kisspeptin at levels corresponding to those found in pregnancy, results in increased differentiation of human naive T cells into T-regulatory cells (19).

The placenta is considered the main source of kisspeptin during pregnancy and the *KISS-1*/*Kiss-1* gene is expressed in syncytiotrophoblasts, whereas its receptor is expressed in both cytotrophoblasts and syncytiotrophoblasts (12) (**Table 1**). Expression of kisspeptin and its receptor is high during early pregnancy and declines as the placenta matures, thus highlighting kisspeptin’s role in placentation (14). Interestingly, circulating kisspeptin levels increase linearly with advancing gestation and kisspeptin-54 immunoreactivity dramatically rises from 1230 pmol/L during the first trimester to 9590 pmol/L during the third trimester and returns to non-pregnant levels (<100 pmol/L) soon after birth (8 pmol/L) (6, 7, 20).

**Table 1:** Summary of Kisspeptin gene, receptor and circulating levels in different pregnancy states.

Pregnancy state	KISS-1 expression	KISS-1 receptor expression	Circulating Kisspeptin levels
**Healthy Pregnancy**	Increased in first trimester (12) - Villous cytotrophoblasts	Increased in first trimester (12) - Villous cytotrophoblasts - Syncytiotrophoblasts - Extravillous cells	Increase linearly with pregnancy progression (20)
**Miscarriage**	Reduced (11) - Trophoblasts	No difference in women with recurrent pregnancy loss (11)	Reduced (20–25)
**Ectopic pregnancy**	Reduced (26) - Embryonic tissue	NA	Reduced (25, 26) No difference (20)
**Preterm Birth**	Increased (27) - Placental tissue	NA	No difference (27, 28) (unadjusted KP higher in late first trimester) (28)
**Foetal Growth Restriction**	NA	NA	Reduced (28–31)
**Pre*-*Eclampsia**	Increased (32–35) Increased (EPE) (36) No difference (LPE) (36) Decreased (37, 38) - Placental tissue	Increased (35, 37) No difference (32) - Placental tissue	Reduced in PE: 1^st^ trimester (29, 39), 2^nd^ trimester (40–42), 3^rd^ trimester (32, 40–43) Reduced in EPE 9-13 wks (28) No difference in PE (36, 44) Increased in LPE 9-13 wks (28) No difference in PIH (40, 44) No difference in HDP: 1^st^ and 2^nd^ trimesters (28) Increased in HDP: 3^rd^ trimester (28)
**Gestational Diabetes**	Increased (35, 45) - Syncytiotrophoblasts - Cytotrophoblasts	Increased (35, 45) - Syncytiotrophoblasts - Cytotrophoblast	No difference (46) Reduced (28, 40, 47)
**Gestational Trophoblastic disease**	Molar pregnancy: No difference (38, 48) Choriocarcinoma: Decreased (38, 48)	Molar pregnancy: No difference (38, 48) Choriocarcinoma: Decreased (38, 48)	Choriocarcinoma: Increased (7)

EPE, early onset pre-eclampsia; HDP, hypertensive disorders of pregnancy; KP, kisspeptin; LPE, late onset pre-eclampsia; NA, not applicable; PE, pre-eclampsia; PIH, pregnancy induced hypertension.

Circulating kisspeptin levels are affected by several variables in healthy pregnancy (20). Whilst gestational and maternal age are associated with raised kisspeptin levels, Afro-Caribbean ethnicity, smoking during pregnancy, and high body mass index (BMI) are associated with reduced kisspeptin levels (20). Additionally, kisspeptin levels have been shown to be lower in serum compared to plasma samples, and are influenced by pre-analytical factors such as collection tube type, processing time and time to sample storage (49).

## Kisspeptin in Pregnancy Complications

### 1. Kisspeptin in Miscarriage

Miscarriage is the spontaneous loss of an intrauterine pregnancy before 24 weeks of gestation and affects 1 in 5 clinical pregnancies (50). Miscarriage predominantly occurs during the first trimester of pregnancy and the majority of early miscarriages are due to a genetic abnormality of the developing embryo, however other causes include endocrine, anatomical, and immunological factors (51).

Miscarriage diagnosis can be challenging as often a pregnancy is failing for a time before pregnancy loss has conclusively been confirmed. This uncertainty can exacerbate the psychological burden related to investigating possible miscarriage, with up to 6% of women suffering from moderate-severe depression, 17% from moderate-severe anxiety and 18% from post-traumatic stress disorder (52). To date, there is no clinical predictor of miscarriage, however recent data demonstrates a potential for kisspeptin as a biomarker of miscarriage.

Kisspeptin levels (adjusted for gestation) are markedly reduced by 60-79% in women with miscarriage compared to healthy pregnancy (20–25) (**Table 2.1**). Above average levels, when corrected for gestational age, are reassuring with a <1% chance of miscarriage (20), whereas kisspeptin levels 95% lower than the median for that gestation are associated with up to an 85% chance of miscarriage. Concordantly, *KISS-1* expression is decreased in the placentae of women with recurrent spontaneous abortion compared to those who undergo voluntary termination of pregnancy (11). Furthermore, whilst kisspeptin’s high diagnostic performance for identifying miscarriage is maintained in late-first trimester pregnancies (>8 weeks of gestation), that of β-human chorionic gonadotrophin (β-hCG) worsens (20). Thus, the combination of both kisspeptin and β-hCG can be used to ensure high diagnostic accuracy at all gestations (AUCROC 0.92, 95% CI 0.89-0.95) (20, 22, 24). Kisspeptin has also been shown to reflect different types of miscarriage, with lower levels reported in complete (no retained products of conception) versus incomplete (retained products of conception) or missed (empty gestational sac or a foetal pole with no heartbeat) miscarriage (20). Additionally, both kisspeptin and β-hCG levels decline with closer proximity to miscarriage confirmation, and therefore repeat measurements every 1-2 weeks could enable further risk-stratification of miscarriage risk in clinical practice (20).

**Table 2:** Circulating Kisspeptin Levels in pregnancy complications.

**2.1. KISSPEPTIN IN MISCARRIAGE**						
**Author**	**Study Design**	**Cohort**	**Sample size**	**Kisspeptin measurement**	**Kisspeptin and βHCG values**	**AUCROC**
**Kavvasoglu (2011)** (21)	Prospective Cohort	Pregnant women who delivered to term and miscarriage	Controls 20 Miscarriage 20	Plasma at 7-18 wks GA KP-10 ELISA (Phoenix, Germany)	Kisspeptin pg/ml (median, min-max)* Controls: 5,783 (3,168–9,953) Miscarriage: 391 (152–951)	NA
**Jayasena** **(2014)** (22)	Prospective Cohort	Asymptomatic pregnant women	Controls 899 Miscarriage 50	Plasma at 7-14 wks GA All KP forms In house RIA	Kisspeptin MoM (mean ± SD)* Controls: 1.06 ± 0.42 Miscarriage: 0.42 ± 0.39 β-hCG MoM (mean ± SD)* Controls: 1.08 ± 0.47 Miscarriage: 0.69 ± 1.35	**KP** 0.899 **βhCG** 0.775
**Mumtaz** **(2017)** (23)	Case-Control	Women with infertility undergoing ICSI treatment	Controls 28 Preclinical abortion 30	Serum before treatment All KP forms ELISA (Kiss-1, China)	Kisspeptin ng/L (mean ± SEM)* Controls: 296.23 ± 12 Miscarriage: 215.11 ± 34.14	
**Sullivan-Pyke (2018)** (24)	Case-Control	Symptomatic pregnant women	Controls 20 Miscarriage 20	Serum at 6-10 wks GA KP-54 ELISA (Peninsula, USA)	Kisspeptin ng/ml (median, IQR)* Controls: 1.50 [0.55 – 3.72] Miscarriage: 0.20 [0.07 – 0.37] β-hCG mIU/mL (median, IQR)* Controls: 117202 [83975 – 148784] Miscarriage: 4739 [1858 – 8650]	**KP** 0.953 **βhCG** 0.994
**Yu** **(2019)** (53)	Case-Control	Women with infertility undergoing IVF/ICSI treatment	Controls 28 Miscarriage 21	Serum at (i) 12 days after blastocyst transfer and (ii) 4 days after pregnancy confirmation All KP forms ELISA (BlueGene, China)	**Kisspeptin** No significant difference between controls and miscarriage β-hCG* Significantly lower in miscarriage compared to controls	**KP** (i) 0.63, (ii) 0.76 **βhCG** (i) 0.76, (ii) 0.89
**Hu** **(2019)** (12)	Case-Control	Women with infertility undergoing frozen thawed embryo transfer	Controls 47 Miscarriage 28	Serum at (i) 14 days and (ii) 21 days after embryo transfer KP-54, KP-10 RIA (Phoenix, USA)	**Kisspeptin pg/ml (mean ± SD)** Controls: (i) 420.9 ± 201.5, (ii) 730.8 ± 274.4 Miscarriage: (i) 434.9 ± 215.1, (ii) 762.2 ± 210.3 β-hCG IU/L (mean ± SD)* Controls: (i) 1791 ± 1730, (ii) 21833 ± 16160 Miscarriage: (i) 777.8 ± 783.8, (ii) 6720 ± 4413	**KP** 0.533 **βhCG** 0.777
**Abbara** **(2021)** (20)	Case-Control	Asymptomatic and Symptomatic pregnant women	Controls 265 Miscarriage 95	Plasma every 2 wks between 6-14 wks GA All KP forms In house RIA	Kisspeptin MoM (median, IQR)* Controls: 1.00 [0.63–1.31] Miscarriage: 0.21 [0.08–0.47] β-hCG MoM (median, IQR)* Controls: 1.00 [0.74–1.32] Miscarriage: 0.30 [0.08–0.64]	**KP** 0.874 **βhCG** 0.859
**Gorkem** **(2021)** (54)	Case-Control	Asymptomatic and Symptomatic pregnant women	Controls 30 Miscarriage 30 Threatened miscarriage 30	Serum at 7-9 wks GA KP-54 ELISA (Cloud-Clone Corp, USA)	**Kisspeptin ng/ml (median, IQR)** Controls: 86.7 [69.5-112.4] Miscarriage: 102.5 [79.5-123.5] Threatened miscarriage: 101.7 [85.4-139.4]	NA
**Yuksel** **(2022)** (25)	Prospective Case-Control	Symptomatic pregnant women with a pre-diagnosis of EP or miscarriage and healthy pregnancy	Controls 23 Miscarriage 23	Serum at 5-6 wks GA KP form unclear ELISA (Mybiosource, USA)	Kisspeptin ng/ml (median, min-max)* Controls: 1.48 (1.29–1.80) Miscarriage: 0.11 (0.08–0.16) β-hCG mIU/ml (median, min-max)* Controls: 6151 (576–19,941) Miscarriage: 1771 (98–11,890)	NA
**2.2. KISSPEPTIN IN ECTOPIC PREGNANCY**						
**Author**	**Study Design**	**Cohort**	**Sample size**	**Kisspeptin measurement**	**Kisspeptin and βHCG values**	**AUCROC**
**Romero-Ruiz** **(2019)** (26)	Prospective Case-Control	Women with normal pregnancy that desired VTOP and EP	VTOP 108 EP 45	Plasma at 4-20 wks GA All KP forms In house RIA	Kisspeptin* Significantly lower in EP compared to controls at all GA stages β-hCG* Significantly lower in EP compared to controls at all GA stages	**KP** 0.909 **βhCG** 0.947
**Abbara** **(2021)** (20)	Case-Control	Asymptomatic and Symptomatic pregnant women	VIUP 42 EP 31 FPUL 82 PPUL 8	Plasma every 2 wks between 6-14 wks GA All KP forms In house RIA	**Kisspeptin pmol/L (mean + SEM)** VIUP: 21.6 ± 41. EP: 20.1 ± 10.6 FPUL: 16.9 ± 12.0. PPUL: 21.5 ± 16.0	NA
**Yuksel** **(2022)** (25)	Prospective Case-Control	Symptomatic pregnant women with a pre-diagnosis of EP or miscarriage and healthy pregnancy	Controls 23 EP 17	Serum at 5-6 wks GA KP form unclear ELISA (Mybiosource, USA)	Kisspeptin ng/ml (median, min-max)* Controls: 1.48 (1.29–1.80). EP: 0.30 (0.22–0.39) β-hCG mIU/ml (median, min-max)* Controls: 6151 (576–19,941). EP: 1333 (94–11,600)	NA
**2.3. KISSPEPTIN IN HYPERTENSIVE DISORDERS OF PREGNANCY AND PRE-ECLAMPSIA**						
**Author**	**Study Design**	**Cohort**	**Sample size**	**Kisspeptin measurement**	**Kisspeptin and βHCG values**	**AUCROC**
**Armstrong** **(2009)** (29)	Retrospective Case-Control	Pregnant women with PE and uncomplicated pregnancies	Controls 317 PE 57	Serum at 16-20 wks GA KP-54 In house ELISA	Kisspeptin pg/ml (median, IQR) * Controls: 1188 [494 – 2298] PE: 1109 [442 – 3903]	NA
**Nijher** **(2010)** (44)	Case-Control	Pregnant women with PE, PIH and uncomplicated pregnancies	Controls 78 PE 9 PIH 78	Plasma at 27-40 wks GA KP-10, KP- 14, KP-54 In house RIA	**Kisspeptin pmol/l (mean ± SE)** Controls: 2878 ± 157 PIH: 2696 ± 299 PE: 3519± 357	NA
**Cetcovic** **(2012)** (40)	Prospective Case-Control	Pregnant women with CH, PIH, PE and uncomplicated pregnancies	Controls 25 CH 22 PIH 18 PE 28 EPE 23 LPE 5	Plasma at (i) 21-25 wks and (ii) 32-36 wks GA KP-10, KP- 14, KP-54 Validated RIA (7)	**Kisspeptin nmol/l (mean ± SD)** Controls: (i) 10.33 ± 2.65, (ii) 20.48 ± 7.60 PE: (i) 4.46 ± 3.73, (ii) 16.03 ± 10.09* CH: (i) 3.42 ± 1.04, (ii0 14.14 ± 10.44 * PIH: (i) 8.46 ± 6.24, (ii) 25.68 ± 9.2	NA
**Madazli** **(2012)** (39)	Retrospective Case-Control	Pregnant women with PE and uncomplicated pregnancies	Controls 30 PE 31	Plasma at 11-14 wks GA KP form unclear ELISA: (Phoenix, Germany)	Kisspeptin pmol/l (mean ± SD) * Controls: 1995 ± 375 PE:1554 ± 385	**KP** 0.797 **PlGF** 0.831
**Adali** **(2012)** (43)	Cross-Sectional	Pregnant women with PE (mPE GA 35.4 ± 0.83*, sPE GA 33.09± 0.75*) and uncomplicated pregnancies (GA 37.66± 0.39)	Controls 50 mPE 15 sPE 24	Plasma at 33-37 wks GA KP-10, KP- 14, KP-54 ELISA (Phoenix, Germany)	Kisspeptin ng/ml (mean± SE) * Controls: 9.69 ± 1.35 mPE: 2.61 ± 0.40 sPE: 1.17 ± 0.24	NA
**Logie** **(2012)** (41)	Cross-Sectional	Lean women with healthy pregnancy (controls) and obese women (BMI >40kg/m^2^) with uncomplicated pregnancy or PE	Controls 39 Obese (uncomplicated) 112 Obese PE 7	Plasma at (i) 16, (ii) 28, (iii) 36 wks GA KP form unclear ELISA (Phoenix, Germany)	**Kisspeptin at 16 wks pM (mean ± SEM)** Lower in obese PE compared to uncomplicated obese and controls*	**KP** (i) 0·80, (ii) 0·56, (iii) 0·66)
**Ziyaraa** **(2015) (** 42)	Prospective Case-Control	Pregnant women who completed GA 20 wks with mild and severe EPE and uncomplicated pregnancies Difference in BMI between the groups *	Controls 40 PE 60 Mild EPE 39 Severe EPE 21	Plasma at (i) 20-27 wks and (ii) 28-40 wks KP-10 ELISA (Phoenix, Germany)	**Kisspeptin ng/ml (mean± SEM)** Controls: (i) 2.30 ± 0.51, (ii) 2.95 ± 1.82 Mild EPE: (i) 2.18 ± 0.76, (ii) 2.16 ± 0.48 * Severe EPE: (i) 1.59 ± 0.26 (1^st^) *, (ii) 2.39 ± 0.57 Mild vs Severe EPE:(i) *, (ii) (NS)	NA
**Matjila** **(2016)** (32)	Case-Control	Patients with (mean GA 32.95 ± 0.53 *) and without EPE (mean GA 38.03 ± 0.06 *) undergoing elective caesarean delivery	Controls 30 EPE 19	Serum at 32-39wks GA KP-10 ELISA (Phoenix, Germany)	Kisspeptin ng/ml (mean± SEM) * Controls: 1.66 ± 0.59 ng/ml PE: 0.58 ± 0.39	NA
**Abbara** **(2022)** (28)	Case-Control	Pregnant women with antenatal complications and uncomplicated pregnancies	Controls 265 HDP 32 PE 20 (EPE, LPE) PIH 12	Plasma at (i) <9, (ii) 9-13, (iii) 14-27, (iv) 28-40 wks GA KP-10, KP-14, KP-54 In-house RIA	**Kisspeptin pmol/L (mean± SEM)** HDP Vs Controls No significant difference in (i), (ii), (iii) Higher in HDP than controls (iv) * LPE Vs Controls No significant difference in (i), (iii), (iv) Higher in LPE than controls(ii) * EPE Vs Control No significant difference in (i), (iii), (iv) Lower in EPE than controls(ii) * Kisspeptin MoM (median) * Higher in HDP than control pregnancies	NA
**2.4. KISSPEPTIN IN GESTATIONAL DIABETES MELLITUS**						
**Author**	**Study Design**	**Cohort**	**Sample size**	**Kisspeptin measurement**	**Kisspeptin and βHCG values**	**AUCROC**
**Cetcovic** **(2012)** (40)	Prospective Case Control	Pregnant with and without a diagnosis of GDM	Controls 25 GDM 20	Plasma at (i) 21-25 and (ii) 32-36 wks GA KP-10, KP- 14, KP-54 Validated RIA (7)	Kisspeptin nmol/l (Mean ± SD) * Controls: (i) 10.33 ± 2.65; (ii) 20.48 ± 7.60 GDM: (i) 4.51 ± 3.18*; (ii) 11.643 ± 7.6 *	NA
**Bowe** **(2019)** (47)	Case-Control	Pregnant women with and without a diagnosis of GDM	Controls 62 GDM 26	Plasma at 26-34 wks GA KP form unclear ELISA (Phoenix, Germany)	Kisspeptin pmol/l (Mean ± SEM) * Controls: 1270.9 ± 67.1 GDM: 889.9 ± 96.6	NA
**Arslan** **(2020)** (46)	Cross-Sectional	Pregnant women with and without a diagnosis of GDM	Controls 82 GDM 76	Serum at 24-28 wks GA KP-54 ELISA (Human KISS-54 kits-Biotek Synergy HT)	**Kisspeptin pmol/l (Mean ± SD)** Controls: 161.3 ± 78.2 GDM: 187.6 ± 132.3 (NS)	NA
**Abbara** **(2022)** (28)	Case-Control	Pregnant women with antenatal complications and uncomplicated pregnancies	Controls 265 GDM 35	Plasma at <9, 9-13, 14-27, 28-40 wks GA KP-10, KP-14, KP-54 In-house RIA	**Kisspeptin nmol/l (Median [IQR])** No difference between control and GDM pregnancies in all trimesters Kisspeptin MoM (median)* GDM lower than control pregnancies	NA
**2.5. KISSPEPTIN IN PRETERM BIRTH**						
**Author**	**Study Design**	**Cohort**	**Sample size**	**Kisspeptin measurement**	**Kisspeptin and βHCG values**	**AUCROC**
**Torricelli** **(2008)** (27)	Observational	Pregnant women delivering at term (GA 38-40 wks, by SVD or ECS) and preterm (GA 32-34 wks)	Term SVD 15 Term ECS 15 Preterm 10	Plasma at delivery All KP forms ELISA (Phoenix, Germany)	**Kisspeptin ng/ml (mean ± SEM)** Term SVD: 4.332 ± 2.10 Term ECS: 4.021 ± 1.67 Preterm: 4.781 ± 1.51	NA
**Abbara** **(2022)** (28)	Case-Control	Pregnant women with uncomplicated pregnancies and preterm birth (GA 24-37wks)	Controls 265 Preterm 11	Plasma at (i) <9, (ii) 9-13, (iii) 14-27, (iv) 28-40 wks GA KP-10, KP-14, KP-54 In-house RIA	Kisspeptin* Adjusted KP higher in PTB than controls in all trimesters Unadjusted KP levels in (ii) higher in PTB than controls	NA
**2.6. KISSPEPTIN IN FOETAL GROWTH RESTRICTION**						
**Author**	**Study Design**	**Cohort**	**Sample size**	**Kisspeptin measurement**	**Kisspeptin and βHCG values**	**AUCROC**
**Smets** **(2008)** (30)	Case-Control	Pregnant women at risk of PE, IUGR and SGA **Birth weight (g)** Controls 3623 ± 334 SGA 2665 ± 369	Controls 31 SGA 31	Plasma at 8-14 wks GA KP-10 Ab RIA (Phoenix, USA)	Kisspeptin pmol/L (mean ± SD)* Controls: 2035 ± 1260 IUGR: 1376 ± 1317 **β-hCG pg/ml (mean ± SD)** Controls: 62 ± 56 IUGR: 61 ± 55	NA
**Armstrong** **(2009)** (29)	Retrospective case-control	Pregnant women with IUGR and uncomplicated pregnancies **Birth weight (g)** Controls 3496 ± 36.6 IUGR 2307 ± 17.4	Controls 317 IUGR 118	Serum at 16-20 wks GA KP-54 In house ELISA	Kisspeptin pg/ml (median, IQR)* Controls: 1188 [494 – 2298] IUGR: 1164 [442 – 3903) **β-hCG MoM (mean ± SEM)** Controls: 0.97 (0.69) [0.20 – 3.19] IUGR: 0.91 (0.74) [0.50 – 3.6]	NA
**Khalil** **(2018)** (31)	Case-Control	Pregnant women with PE&IGUR, IUGR and uncomplicated pregnancies that underwent ECS **Birth weight (g)** Controls 3300 ± 110 PE&IUGR 2180 ± 220 IUGR 2280 ± 350	Controls 10 PE&IUGR 10 IUGR 10	Serum at 34-38wks GA KP-10 ELISA (Life span Biosciences)	Kisspeptin ng/ml (mean ± SD)* Controls: 2900 ± 600 PE&IUGR: 1640 ± 400 IUGR: 1630 ± 300	NA
**Abbara** **(2022)** (28)	Case-Control	Pregnant women with antenatal complications and uncomplicated pregnancies	Controls 265 FGR 17	Plasma at (i) <9, (ii) 9-13, (iii) 14-27, (iv) 28-40 wks GA KP-10, KP-14, KP-54 In-house RIA	Kisspeptin* Adjusted KP lower in FGR than controls in all trimesters Unadjusted KP levels in (ii) and (iv) lower in FGR than controls	NA
**2.7. KISSPEPTIN IN GESTATIONAL TROPHOBLASTIC DISEASE**						
**Author**	**Study Design**	**Cohort**	**Sample size**	**Kisspeptin measurement**	**Kisspeptin and βHCG values**	**AUCROC**
**Dhillo** **(2006)** (7)	Case-Control	Healthy pregnant women and women diagnosed with invasive mole undergoing chemotherapy	Controls 26 Invasive mole 11	Plasma at (i) 10 wks GA, (ii) 38 wks GA and (iii) 15 days postpartum and (iv) pre and post chemotherapy for invasive mole KP-10, KP-14, KP-54 In-house RIA	Kisspeptin pmol/l (mean ± SE)* Controls 10 wks: 803 ± 13 38 wks: 2,483 ± 302 15 days postpartum: <2 Invasive Mole Pre-chemo: 1,363 ± 1,076 pmol* Post-chemo: <2 β-hCG U/l (mean ± SE) * Controls 10 wks: 72,053 ± 10,936 38 wks: 28,818 ± 11,348 Invasive Mole Pre-chemo: 227,191 ± 152,354 Post-chemo: **<**2	NA

BMI, body mass index; CH, chronic pre-existing hypertension; ECS, elective caesarean section; ELISA, enzyme-linked immunosorbent assay; EP, ectopic pregnancy; EPE, early onset pre-eclampsia; FGR, foetal growth retardation; FPUL, failed (negative pregnancy test 2 weeks from follow-up) pregnancy of unknown location; GA, gestational age; GDM, gestational diabetes mellitus; GTD, gestational trophoblastic disease; HDP, hypertensive disorders of pregnancy; ICSI, intracytoplasmic sperm injection; IQR, interquartile range; IUGR, intrauterine growth retardation; IVF, in vitro fertilisation; KP, kisspeptin; LPE, late onset pre-eclampsia; mPE, mild pre-eclampsia; MoM, multiple of the median; NA, not applicable; NS, no statistically significant difference; PE, pre-eclampsia; PIH, pregnancy induced hypertension; PlGF, placenta growth factor; PPUL, persistent (more than three static serial βhCG levels) pregnancy of unknown location; RIA, radioimmunoassay; SD, standard deviation; SEM, standard error of the mean; SGA, small for gestational age baby; sPE, severe pre-eclampsia; SVD; spontaneous vaginal delivery; VIUP, intrauterine pregnancy viable at 12 weeks’ gestation; VTOP, voluntary termination of pregnancy; wks, weeks.

*p-values indicate statistically significant difference.

Studies involving women with infertility who undergo assisted reproductive techniques (*in vitro* fertilisation, intracytoplasmic sperm insemination (ICSI) or frozen thawed embryo transfer) have found reduced β-hCG levels in miscarriage compared to controls, but no difference in kisspeptin levels (53, 55). These findings may be due to the very early gestations at which kisspeptin levels were assessed (2-3 weeks following, or even before, pregnancy confirmation) (53, 55). Indeed, kisspeptin may not be expressed in the placenta at high levels prior to 6 weeks of gestation, suggesting that β-hCG levels may be more useful at these very early gestations (26).

### 2. Kisspeptin in Ectopic Pregnancy

Ectopic pregnancy (EP) affects 2% of pregnancies and occurs when a fertilised ovum implants and develops outside the uterine cavity, most commonly within the fallopian tube (56). EP can result in tubal rupture and accounts for 9-13% of all pregnancy-related deaths in developed countries and can compromise a woman’s future fertility (57). EP is currently diagnosed by serial β-hCG measurements in combination with ultrasound, although laparoscopy is often required to provide a definitive diagnosis (58). The sensitivity and specificity of these tests significantly decrease in the case of pregnancies of unknown location (PUL) as false positive or negative diagnoses may occur. This is important as an incorrect diagnosis may lead to termination of a healthy pregnancy (59). Accordingly, different biomarkers have been investigated in an attempt to improve the diagnostic accuracy of EP, including kisspeptin.

Some studies have found that kisspeptin levels in EP are lower than in healthy pregnancy but higher than in miscarriage (25, 26). However, another study demonstrated that kisspeptin levels are not significantly altered between women with viable intrauterine pregnancies (VIUPs) and those with either EP or failing or persistent PUL, after adjusting for confounding variables (20) (**Table 2.2**). Current evidence remains limited, and larger studies are required to determine kisspeptin’s performance as a diagnostic marker in EP at early gestations (<6 weeks).

### 3. Kisspeptin in Hypertensive Disorders of Pregnancy and Pre-Eclampsia

Hypertensive disorders affect 5% of all pregnancies (60) and include pre-existing chronic hypertension (CH), pregnancy induced hypertension (PIH) and pre-eclampsia (PE). PIH is defined as new onset hypertension (BP ≥140/90mmHg) occurring after 20 weeks of gestation, PE is PIH with proteinuria (urine >3g/24 hours) or significant end-organ dysfunction, and severe PE is the presence of at least one of: hypertension (BP≥160/110 mmHg), visual disturbance, chest pain, dyspnoea, pulmonary oedema, seizures, or neonatal distress (61). PE is further classified, according to the onset of clinical features, into early-onset PE (EPE <34 weeks of gestation) and late-onset PE (LPE ≥34 weeks of gestation). EPE is associated with impaired trophoblast invasion, defective spiral artery remodelling and adverse perinatal complications including IUGR (62). LPE occurs due to hypoxic stress and impaired perfusion but is less likely to compromise foetal growth (63, 64). Currently, PE diagnosis is based on early pregnancy risk factor screening, uterine artery Doppler velocimetry and biomarkers such as PPAP-A or placental growth factor (PlGF) (61). Kisspeptin has been implicated in the pathogenesis of PE through reduced angiogenesis, decreased cytotrophoblast invasion and increased trophoblast apoptosis, and thus could have potential in predicting PE (16–18).

Levels of circulating kisspeptin in HDP vary in the literature, and largely differ according to HDP subtype, severity, and onset (**Table 2.3**). Most of the studies report reduced circulating kisspeptin levels in PE compared to normotensive pregnant controls (29, 32, 40–43, 65) and therefore kisspeptin is considered to reflect placental dysfunction. However, expression of *KISS-1*, which inhibits trophoblast invasion and results in defective transformation of the spiral arteries, is increased in the placentae of PE pregnancies, thus supporting its role in the pathophysiology of PE (32–34, 36, 66) (**Table 1**). Nonetheless, there are also some reports of decreased *KISS-1* expression in PE placentae (37, 38) (**Table 1**). Furthermore, evidence suggests that circulating kisspeptin levels decline as the severity of PE increases, which could also reflect reduced placental mass in more severe disease. Indeed, both circulating kisspeptin levels and placental mass is reduced in EPE compared to LPE (28, 42, 67). Additionally, pregnant women with pre-existing hypertension and PE, states associated with a higher burden of disease, have reduced kisspeptin levels compared to PIH (40).

Whilst most studies demonstrate reduced kisspeptin levels in PE, a recent study found that kisspeptin levels are increased in HDP during the third trimester of pregnancy (**Table 2.3**). However, there was no association between circulating kisspeptin levels and severity of PET (28). It is likely that complexity in the categorisation, severity, and onset of PET, and the need for correction for possible confounders such as BMI and gestational age, could explain differences between kisspeptin levels observed in the current studies. Larger observational studies that are carefully designed to address these and look at each PET-subset throughout pregnancy would therefore be valuable in resolving these inconsistencies.

### 4. Kisspeptin in Gestational Diabetes Mellitus

During pregnancy a physiological rise in maternal insulin resistance provides glucose to the developing foetus (68, 69). This insulin resistance leads to maternal pancreatic β-cell adaptation and increased insulin secretion. Failure of these changes results in gestational diabetes mellitus (GDM), which affects up to 20% of pregnancies worldwide (70).

Kisspeptin receptors are expressed in pancreatic β-cells (71) and have been implicated in β-cell adaptation during pregnancy. Exogenous kisspeptin administration has variable physiological effects on the glucose-dependent regulation of pancreatic beta-cells. For instance, KISS-1 peptide (KP-145) (71), KP-13 (72), KP-10 (72–74) potentiates glucose-stimulated insulin secretion (GSIS) in animal and human islets *in-vitro*. KP-54 increases GSIS in healthy men following an intravenous glucose tolerance test (IVGTT), which induces high glucose levels (75). On the other hand, Vikam and colleagues have found that KP-13 and KP-54 drives dose-dependent inhibitory effects on insulin secretion in mouse islets in the presence of lower glucose concentrations (2.8-11.1 mmol/l), compared to controls, which is not observed at higher glucose concentrations (76). Furthermore, chronic administration of KP-10 in non-pregnant mice enhances GSIS and improves glucose tolerance (47). Interestingly, hyperlipidaemia, impaired glucose tolerance (IGT) and weight gain develops in *Kiss-1r*-null female mice exclusively, thus suggesting sexual dimorphism in kisspeptin’s effects on metabolism and glucose homeostasis (77).

In late gestation murine pregnancy, β-cell specific *Kiss-1r*-knockout models and pharmacological inhibition of *Kiss-1r* leads to reduced GSIS and development of IGT, which is not observed in non-pregnant states or wild-type controls (47). This supports a role for β-cell kisspeptin signalling in the regulation of glucose homeostasis during pregnancy. Loss of kisspeptin signalling in the ß-cell-specific *Kiss-1r*-knockout models also attenuates the increased ß-cell proliferation normally seen during murine pregnancy when assessed with bromodeoxyuridine (BrdU) labelling. Nonetheless, the levels are not reduced to non-pregnant levels, suggesting contribution of other signals in pancreatic β-cell proliferation during pregnancy (47, 78).

In human pregnancies with GDM, placental *KISS-1* and *KISS-1R* expression is elevated in the third trimester (35, 45) (**Table 1**), whereas circulating kisspeptin levels have been either lower (40, 47) or not significantly altered (28, 46) (**Table 2.4**). Finally, Bowe and colleagues have demonstrated a positive correlation between third trimester kisspeptin levels and oral glucose–stimulated insulin levels at 60 minutes (r^2 =^ 0.18; P < 0.0001) and AUC serum insulin over the OGTT (r^2^ = 0.13; P=0.0013) in women with GDM (47).

### 5. Kisspeptin in Pre-Term Birth

Pre-term birth (PTB) is defined as delivery prior to 37 weeks of gestation and affects 11% of pregnancies (79, 80). Kisspeptin has been proposed to initiate labour through increased oxytocin neuronal firing rate in pregnant rats and thus may play a potential role in PTB (81). Gestation adjusted kisspeptin levels are higher in PTB-affected pregnancies than in control pregnancies during the late-first trimester, with the adjusted odds of PTB being increased by 20% (95% CI, 1-42%) for every 1 nmol/L increase in plasma kisspeptin (28) (**Table 2.5**). Furthermore, *KISS-1* mRNA expression is higher in preterm placentae than in term placentae delivered vaginally or by Caesarean section thus indicating that increased kisspeptin expression could be involved in the induction of labour (27) (**Table 1**)W. However, no alteration in circulating kisspeptin levels have been reported to date during the third trimester between healthy pregnancy and PTB and thus more data is needed to elucidate whether there are changes in kisspeptin levels preceding and around the time of spontaneous labour (27, 28).

### 6. Kisspeptin in Foetal Growth Restriction

Foetal growth restriction (FGR) encompasses both intrauterine growth restriction (IUGR, foetal weight <10^th^ centile for gestational age with abnormal umbilical artery doppler results) and small for gestation age (SGA, delivery weight <10^th^ percentile for gestational age) (82, 83). FGR is thought to arise from abnormal trophoblast invasion and spiral artery remodelling that limits oxygen supply to the placenta (84, 85). The resulting ischemic injury generates reactive oxygen species which lead to apoptosis and restriction of placental and foetal growth (84, 85). To date, four studies have demonstrated significantly reduced kisspeptin levels in FGR versus healthy pregnancy in all three trimesters (28–31) (**Table 2.6**). Thus, low circulating kisspeptin levels could reflect low placental mass in pregnancies affected by FGR.

### 7. Kisspeptin in Gestational Trophoblastic Disease

Gestational trophoblastic disease (GTD) is characterised by an abnormal proliferation of placental tissue and comprises of choriocarcinoma, invasive mole, placental site trophoblastic tumour and epithelioid trophoblastic tumour (86). Molar pregnancy is a benign form of GTD, whereas choriocarcinomas are more aggressive, however both exhibit high β-hCG levels and respond well to chemotherapy (87). Serum β-hCG measurement aids with GTD diagnosis, staging and prognostication before and after chemotherapy (88).

*KISS-1* and *KISS-1R* expression is significantly lower in malignant choriocarcinoma cells compared to molar and healthy pregnancies (38, 48) (**Table 1**). Conversely, circulating kisspeptin levels are elevated in malignant GTD compared to healthy pregnancies but significantly decline following chemotherapy (7) (**Table 2.7**). The increased circulating kisspeptin levels could reflect an increased malignant trophoblast mass rather than an elevation in cellular *KISS-1* expression (89). Thus, kisspeptin levels can be altered in choriocarcinomas and other GTDs, which is interesting when considering the original identification of *KISS-1* as an anti-metastatic gene.

## Conclusion

Kisspeptin levels are markedly reduced in miscarriage; and whilst the performance of kisspeptin levels to identify women at high risk of miscarriage is maintained throughout the first trimester, that of β-hCG falls during the latter part of the first trimester. Nevertheless, kisspeptin levels are only mildly elevated at early gestations (< 6 weeks) and therefore can be difficult to detect using current collection and assay methods. Thus, measuring kisspeptin in combination with β-hCG levels could potentially overcome this deficiency at early gestations. Due to the current difficulty in miscarriage diagnosis and the lack of available biomarkers, the high performance of plasma kisspeptin suggests that it has significant potential for further development in this context. Given that kisspeptin has been proposed as a biomarker of healthy placentation, it could potentially be used to recognise late pregnancy complications characterised by abnormal placentation during the first trimester. Regarding HDP, most studies have suggested lower circulating kisspeptin levels but increased placental kisspeptin expression. Kisspeptin levels in pregnancy complications such as PE are confounded by factors such as BMI, disease severity, time of onset, and concomitant FGR, and thus could limit the use of kisspeptin diagnostically.

Overall, current evidence suggests that circulating kisspeptin levels are consistently reduced in miscarriage, EP, FGR, GDM, and increased in PTB and GTD. Larger datasets with adequately sized control cohorts that accurately adjust for gestation, BMI, ethnicity, detailed disease severity phenotype and onset are needed to enable more precise characterisation of the utility of kisspeptin levels in these settings. In summary, circulating kisspeptin is a promising biomarker for early pregnancy loss and further research is needed to assess its potential in other pregnancy complications.

## Author Contributions

BP, JT wrote the manuscript, designed the figures and tables. AA, WSD, ANC reviewed and edited the manuscript and are the corresponding authors. All authors have made a substantial, direct and intellectual contribution to the work and approved the manuscript prior to its submission.

## Funding

This work was supported by grants from the National Institute of Health Research (NIHR), the NIHR/Wellcome Trust Imperial Clinical Research Facility, and the NIHR Imperial Biomedical Research Centre. The Section of Endocrinology and Investigative Medicine was funded by grants from the Medical Research Council (MRC), Biotechnology and Biological Sciences Research Council (BBSRC), NIHR and was supported by the NIHR Biomedical Research Centre Funding Scheme. The views expressed are those of the authors and not necessarily those of the MRC, BBSRC, the NHS, the NIHR, or the Department of Health. BP is supported by an MRC Clinical Training Research Fellowship (Grant Ref: MR/W024144/1). AC is supported by the National Health Service. WD is supported by an NIHR Senior Investigator Award (NIHR RP-2014-05-001). AA is supported by an NIHR Clinician Scientist Award (No. CS-2018-18-ST2-002).

## Conflict of Interest

The authors declare that the research was conducted in the absence of any commercial or financial relationships that could be construed as a potential conflict of interest.

## Publisher’s Note

All claims expressed in this article are solely those of the authors and do not necessarily represent those of their affiliated organizations, or those of the publisher, the editors and the reviewers. Any product that may be evaluated in this article, or claim that may be made by its manufacturer, is not guaranteed or endorsed by the publisher.
